# Mitofusin-2: A New Mediator of Pathological Cell Proliferation

**DOI:** 10.3389/fcell.2021.647631

**Published:** 2021-04-01

**Authors:** Yanguo Xin, Junli Li, Wenchao Wu, Xiaojing Liu

**Affiliations:** ^1^Department of Cardiology, West China Hospital, Sichuan University, Chengdu, China; ^2^Laboratory of Cardiovascular Diseases, Regenerative Medicine Research Center, West China Hospital, Sichuan University, Chengdu, China

**Keywords:** mitochondria, Mfn-2, cell proliferation, target, apoptosis

## Abstract

Cell proliferation is an important cellular process for physiological tissue homeostasis and remodeling. The mechanisms of cell proliferation in response to pathological stresses are not fully understood. Mitochondria are highly dynamic organelles whose shape, number, and biological functions are modulated by mitochondrial dynamics, including fusion and fission. Mitofusin-2 (Mfn-2) is an essential GTPase-related mitochondrial dynamics protein for maintaining mitochondrial network and bioenergetics. A growing body of evidence indicates that Mfn-2 has a potential role in regulating cell proliferation in various cell types. Here we review these new functions of Mfn-2, highlighting its crucial role in several signaling pathways during the process of pathological cell proliferation. We conclude that Mfn-2 could be a new mediator of pathological cell proliferation and a potential therapeutic target.

## Introduction

Cell proliferation is a precision–control process, which is essential for embryonic and postnatal development (Takeuchi and Nakamura, 2014). Under pathological conditions, abnormal cell proliferation is a central mechanism attributing to disease progressions. Abnormal cell proliferation includes both abnormal cell division and abnormal cell differentiation (Fajas, 2003). These processes are common in various diseases. For example, cardiac fibroblast proliferation and fibroblast-to-myofibroblast transition resulted in cardiac fibrosis (Nagpal et al., 2016), a pathological process characterized by the accumulation of extracellular matrix in the cardiac interstitium. Abnormal hepatic stellate cell (HSCs) trans-differentiation, activation, and proliferation of HSCs are the primary driving force to promote chronic cholestatic liver diseases and facilitate the progression of liver fibrosis (Liu et al., 2019). Proliferation is also a main characteristic of cancer cells and the base of metastasis (Carmeliet et al., 1998). Therefore, a thorough understanding of the underlying mechanisms regulating various pathological cell proliferations is the premise for developing new therapeutic strategies.

**FIGURE 1:**
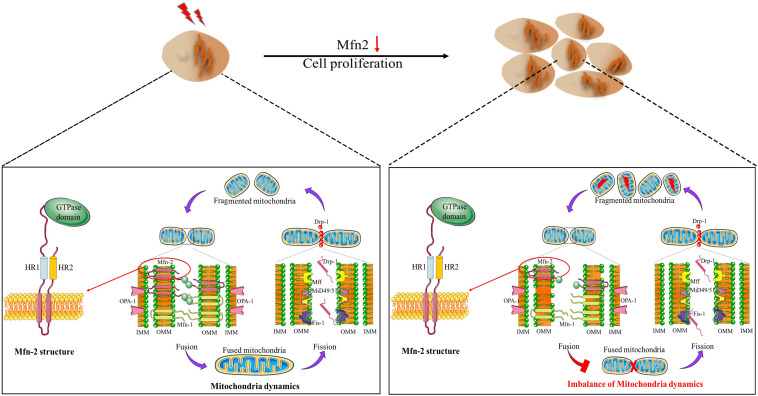
Mitochondria dynamics and Mfn-2 structure. Mfn-2 is one of the main factors regulating mitochondrial fusion. The N-terminal of Mfn-2 is the GTPase domain. There are two HR domains and two transmembrane domains.

Previously, mitochondria have been regarded as static and isolated organelles. More recently, they are found to undergo constant changes in morphology, including fission, fusion, and network formation, and they can also relocate to different parts in cells (trafficking); all of these processes are termed mitochondrial dynamics. Mitochondrial dynamics is essential to maintain the normal function of cells such as energy metabolism, calcium homeostasis, and reactive oxygen species generation (Vafai and Mootha, 2012; Tsushima et al., 2018). Mitochondria dynamics (Figure 1) is closely related to cell proliferation. Previous evidence has set a link between mitochondria dynamics and cell proliferation; high levels of mitochondrial fission are associated with active proliferation (Chen and Chan, 2017). Mitra et al. (2009) reported that maintaining mitochondria in hyperfused morphology could regulate the cell transition from G1 to S phase. In addition, the number of mitochondria throughout the cell cycle is mostly constant, which is attributed to mitochondria dynamics to a great extent (Carlton et al., 2020). Oncocytes could alter mitochondria dynamics to support their proliferation property (Senft and Ronai, 2016).

Research in the past two decades or so have revealed that the mechanisms of mitochondrial dynamics are regulated by a group of highly conserved guanosine triphosphatases (GTPase) (Misaka et al., 2002; Rojo et al., 2002; Son et al., 2017). Mitofusin 1 (Mfn-1) and mitofusin 2 (Mfn-2) control the fusion of the outer mitochondrial membrane (OMM), while optic atrophy 1 (OPA1) regulates the fusion of the inner mitochondrial membrane. Mitochondrial fission is under-controlled by the dynamic-related protein 1 (Drp1), which interacts with several OMM proteins, such as Mid51 and Mid49. Mfns are outer mitochondria membrane proteins, with GTPase domain in the N-terminal, followed by a hydrophobic heptad repeat (HR1), the transmembrane anchor, and HR2. Mfns anchored to OMM by the C-terminal binding domain, and the N-terminal domain extruded to the cytoplasm (Fritz et al., 2001; Rojo et al., 2002; Figure 1). Mammalian Mfn-1 and Mfn-2 shared about 80% similarity, consisting of 737 and 757 amino acids, respectively. However, Mfn-1 exhibited higher GTPase activity than Mfn-2, while Mfn-2 exhibited higher GTP affinity. It is reported that Mfn-1 functions in mitochondria tethering, while Mfn-2 has other distinct functions in addition to the fusion reaction (Ishihara et al., 2004). For example, Mfn-2 has been reported to be involved in PINK/Parkin-mediated mitophagy process (Chen and Dorn, 2013). Mfn-2 is one of the first proteins identified to mediate the tethering of endoplasmic reticulum (ER) and mitochondria in mammals (Vance, 1990). This domain, named mitochondria-associated membranes (MAM), has been the new frontiers in bioenergetics and biophysical studies of intracellular organelles. ER lost its physiological morphology, and the ER–mitochondria interaction was disrupted after Mfn-2 ablation (de Brito and Scorrano, 2008). Increasing evidence has indicated that MAM is closely related to cell proliferation in various diseases (Wang et al., 2009; Danese et al., 2017; Yang et al., 2019). Comparing with Mfn-1, Mfn-2 may play a vital role in cell proliferation *via* various pathways.

In this review, we summarized recent advancements in the study of mitochondrial fusion protein Mfn-2. We focus on the link between altered Mfn-2 and pathological cell proliferation.

## Overview of Mitofusin-2 and Cell Proliferation

**FIGURE 2:**
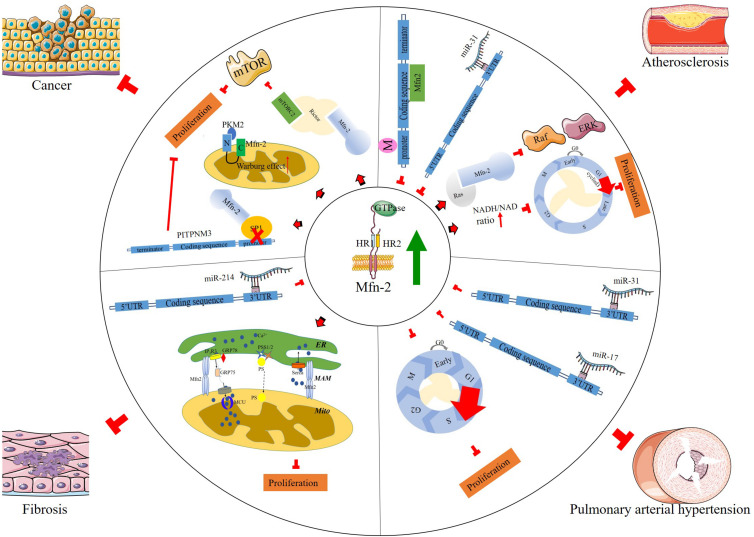
Signaling pathways mediate cell proliferation in various diseases.

Current evidence indicates that Mfn-2 plays various roles in many cellular processes, including cell proliferation and cell death (Chen et al., 2004; Wang et al., 2010; Zhao et al., 2012). Chen et al. (2004) found that Mfn-2 expression decreased in proliferative vascular smooth muscle cells (VSMCs), and overexpression of Mfn-2 could inhibit this proliferation process. Mfn-2 dysfunction has been associated with a variety of pathological conditions, including diabetes mellitus (Hernández-Alvarez et al., 2010), obesity (Bach et al., 2003), Charcot–Marie–Tooth disease (Züchner et al., 2004; Misko et al., 2012), atherosclerosis, hypertension (Chen et al., 2004), and cancer (Zhang et al., 2013; Xu et al., 2017). Collectively, the results of these studies have depicted Mfn-2 as a hyperplasia suppressor gene. In addition, energy is required in cell proliferation; in G1/S phase, mitochondrial elongation requires a higher amount of ATP to sustain cell duplication (Mitra et al., 2009). Mitochondria with intact structure are vital to supply enough ATP in this process, and Mfn-2 is necessary to maintain mitochondrial membrane potential, cellular oxygen consumption, etc. According to Sara’s research (Pich et al., 2005), the role of Mfn-2 in mitochondrial nutrient oxidation is a fusion-independent function. Mitochondria fusion is also necessary to maintain the stoichiometry of the protein components of mtDNA replisome. Mfn-2 mutation could cause diseases due to mtDNA depletion, disrupting mouse embryo fibroblast (MEF) proliferation and postnatal heart development (Silva Ramos et al., 2019). Here a more detailed discussion of how Mfn-2 regulates cell proliferation is provided (Figure 2).

## Mitofusin-2 and Atherosclerosis

Atherosclerosis is the main cause of cardiovascular diseases. It has been clearly demonstrated that the development and progression of atherosclerosis involve inflammation, abnormal proliferation of VSMCs, remodeling of the vascular wall, and evolution of occlusive plaques.

It is reported that the antiproliferative effect of Mfn-2 in serum-evoked VSMC proliferation was mediated *via* the inhibition of Erk/mitogen-activated protein kinase (Erk/MAPKs) signaling followed by cell cycle arrest. Cell transition from G0/G1 phase to S phase is one of the important characteristics for proliferation. EdU assay indicated that downregulation of Mfn-2 resulted in a significant decrease of G0/G1 phase cells and a synchronous increase of the percentage of S-phase cells in homocysteine-induced VSMCs, and overexpression of Mfn-2 obtained the contrary results, suggesting that cell cycle arrest at G0/G1 phase is responsible for the anti-proliferation effects of Mfn-2 (Xu et al., 2019). Two related mechanisms involved in this process were reported: one is that Mfn-2 could bind and inhibit the proto-oncogene Ras directly *via* its p21 Ras-binding domain in the N-terminal to control cell proliferation (de Brito and Scorrano, 2009), while Mfn-2 mutant lacking amino acids 77–91 failed to pull down Ras, with no effect on its function in the regulation of mitochondria fusion (de Brito and Scorrano, 2009); the other is that Mfn-2 regulates NAD/NADH ratio to control the transition of cell phase (Li et al., 2015). Infection with Mfn-2 adenovirus could increase NADH level and reduce NAD level, blocking the cell cycle at G0/G1 phase.

The accumulation of cholesterol-containing low-density lipoproteins in the intima and endothelium promotes the recruitment of monocytes, which then differentiate into macrophages and initiate inflammatory activation. Inflammation cells release various cytokines and growth factors, the most powerful factors, such as platelet-derived growth factor (PDGF) and its receptors, inducing VSMCs to transform from quiescent state to secretion and proliferation state *via* many signaling pathways, such as MAPK, PI3K/Akt, etc. (Wang et al., 2018). One remarkable characteristic of this process is migration of VSMCs from the media to the intima, contributing to neo-intima formation. In addition, various inhibitors in the process of inflammation could inhibit VSMC proliferation (Uhrin et al., 2018). In Feng’s study, Mfn-2 expression was downregulated in proliferative VSMC primary cells treated with PDGF. Down-regulated Mfn-2 promotes cell proliferation and migration rate not only in PDGF-induced VSMCs but also in arterial smooth muscle cells (ASMCs) mediated by microRNA (miRNA) miR-31 (Huang et al., 2018), which targets the 3′UTR of Mfn-2 and downregulates its expression.

In patients diagnosed with type 2 diabetes, the incidence of atherosclerosis is high, and the expression of Mfn-2 in the muscle decreased; similar results are also observed in high-fat-diet mice. In addition, increasing the level of Mfn-2 could alleviate insulin resistance (Gan et al., 2013) through increasing the phosphorylation levels of PI3K-P85 and AKT2 insulin signaling pathways. Previous studies have identified that nuclear receptor superfamily member peroxisome proliferator-activated receptor-γ (PPARγ) is involved in atheromatous inflammation through the extracellular signal-regulated ERK/MAPKs and p38/MAPK pathways (Chen et al., 2004; Monsalve et al., 2013; Nagy et al., 2013; Yang et al., 2012). Liu et al. (2014) found that Mfn-2 inhibited atherosclerotic plaque formation by increasing PPARγ, and this process may be partially regulated by inactivation of the ERK1/2 and p38/MAPKs pathways. Xu et al. (2019) showed that, in homocysteine (Hcy)-induced atherosclerosis, c-Myc expression was increased, and it bound DNMT1 promoter to cause DNA hypermethylation of the Mfn-2 promoter, resulting in aberrant Mfn-2 transcription that led to VSMC proliferation.

As mentioned above, Mfn-2 is crucial in maintaining MAM structure. MAM is the central platform for the initiation of mitophagy and the formation of autophagosome (Hailey et al., 2010; Hamasaki et al., 2013). Chen and Dorn (2013) has linked Mfn-2 with PINK-Parkin-mediated senescent mitochondria elimination. In their model, PINK phosphorylates Mfn-2 to recruit Parkin to mitochondria, and Parkin, in turn, mediates Mfn-2 ubiquitination and initiates mitophagy. The effects of mitophagy on VSMC survival in response to stimuli in the pathogenesis of vascular disorders have been investigated in numerous studies (Culley and Chan, 2018; Marshall et al., 2018). He et al. (2019) reported that apelin-13 could induce VSMC proliferation and exacerbate atherosclerosis. PINK1/Parkin-mediated mitophagy could promote this process. In addition, a study on miR-145 found that the autophagy level was increased in the carotid intima hyperplasia in C57BL/6J mice (Wang et al., 2020), and activation of autophagy could stimulate aortic VSMC proliferation in metabolic hypertension rats (Wen et al., 2019). Although there is no direct evidence that Mfn-2 regulates cell proliferation *via* autophagy, considering the role of Mfn-2 in the maintenance of MAM, more studies are required to provide evidence linking Mfn-2-mediated autophagy with VSMC proliferation in the future.

## MFN-2 and Pulmonary Hypertension

Pulmonary hypertension is another pathological condition attributed to VSMC proliferation. Pathophysiological changes involve all three layers of pulmonary arteries during vascular remodeling. For instance, endothelial angiogenesis, smooth muscle cell hyperplasia and hypertrophy, adventitial fibroblast proliferation, myofibroblast differentiation, and extracellular matrix deposition have all been reported (Howell et al., 2003; Wilkins et al., 2015). The excessive proliferation and impaired apoptosis of pulmonary artery smooth muscle cells (PASMCs) contribute to vascular obstruction in pulmonary hypertension patients.

The role of Mfn-2 in pulmonary hypertension is complicated. Zhang et al. (2012) reported that hypoxia induced Mfn-2 expression and knock-down Mfn-2 suppressed hypoxia-induced PASMCs proliferation through the PI3K/Akt signaling pathway. However, other studies indicated that the expression of Mfn-2 was decreased in pulmonary hypertension models, and increasing the Mfn-2 level may be a therapeutic strategy (Ryan et al., 2013, 2015; Fang et al., 2016; Lu et al., 2016). Deletion of thrombospondin motifs 8 (ADAMTS8), a secreted protein specifically expressed in the lung and the heart, reduced PASMC proliferation with Mfn-2 upregulation and mitochondrial function improvement (Omura et al., 2019). Downregulation of Mfn-2 may cause more cells to enter the S + G2/M phase of the cell cycle and inhibit the mitochondrial apoptosis pathway. These effects were reversed in PASMCs by Mfn-2 overexpression (Roy et al., 2009), indicating that Mfn-2 could be a therapeutic target in pulmonary hypertension (Ryan et al., 2013). While these discrepancies need to be resolved in future studies, it is clear that Mfn-2 is a critical regulator for pathological pulmonary hypertension.

Recent studies revealed that miRNAs play an important role in the pathogenesis of pulmonary hypertension by regulating ASMC proliferation (Thum et al., 2008; Roy et al., 2009; Bauersachs, 2010; Castoldi et al., 2012; Chen et al., 2014). Several miRNAs have been identified to participate in fibrosis and smooth muscle cell proliferation *via* targeting Mfn-2 directly. Huang et al. (2018) reported that the expression level of miR-31 was increased significantly in the arterial walls of patients with atherosclerosis obliterans. In addition, miR-31 promoted the proliferation and migration of human arterial smooth muscle cells, at least partially, through directly interacting with Mfn-2. In another study, Lu et al. (2016) revealed that miR-17 was upregulated in human PASMCs from patients with pulmonary artery hypertension, and the function of miR-17 in PASMC proliferation and apoptosis was partially mediated by downregulation of Mfn-2.

## Mitofusin-2 and Fibroproliferative Diseases

Fibroblasts are a crucial component of connective tissues in various organs. Fibroblast activation and proliferation is characterized by an excessive accumulation of fibrous connective tissues in response to various stimuli (Kendall and Feghali-Bostwick, 2014). Fibrosis is a key pathological process in the development of various fibroproliferative diseases, such as cardiovascular fibrosis, pulmonary fibrosis, liver cirrhosis, systemic sclerosis, and kidney fibrosis. Fibroblasts produce the structural proteins of extracellular matrix (ECM), such as fibrous collagen (Kendall and Feghali-Bostwick, 2014). Fibroblast activation and proliferation may cause the accumulation of ECM’s components, ultimately leading to disruption of the architecture in various tissues and dysfunction of the organs, such as end-stage liver disease and kidney failure. Mfn-2 could inhibit non-alcoholic fatty liver by interacting with phosphatidylserine (PS) directly. Mfn-2 deficiency could reduce PS transfer from the ER to the mitochondria, inducing mitochondrial dysfunction (Hernandez-Alvarez et al., 2019). Knockdown of Mfn-2 could rescue mitochondria Ca^2+^ transfer and inhibit cell proliferation in kidney cysts (Kuo et al., 2019). In addition, overexpressed Mfn-2 could alleviate glomerular mesangial cell proliferation *via* the MAPK/ERK and PI3K/Akt pathways (Wan-Xin et al., 2012; Chen et al., 2019). Mfn-2 regulates mitochondria fusion and intracellular lipid metabolism, which are tightly linked with lung fibrosis (Chung et al., 2019). Prediabetes is a common pathological condition characterized by increased ventricular mass and wall thickness, in which fibroblast proliferation is one of the key factors. Although this is a complicated biological process, Mfn-2 is considered to play a vital role in this process (Koncsos et al., 2016).

Endoplasmic reticulum stress (ERS) is a cellular state in which the protein folding capacity of ER is overwhelmed due to increased protein load or disruption of the protein folding environment (Berridge, 2002). It has been reported that ERS is involved in fibroblast activation and proliferation in many organs such as the liver (Shin et al., 2013), heart (Spitler and Webb, 2014), kidney (Chiang et al., 2011), and lung (Baek et al., 2012). Previous reports also identified the relationship between Mfn-2 and ERS. For example, in cardiac fibrosis, we found that the expression of Mfn-2 was decreased, and upregulating Mfn-2 inhibited fibroblast proliferation *via* the p-PERK/ATF4 pathway but not the IREα/Xbp1s or c-ATF6 signaling pathway (Xin et al., 2019). Ngoh et al. (2012) and Muñoz et al. (2013) also reported that Mfn-2 was an ERS-inducible protein. Philippe and Gary wrote a review discussing the role of Mfn-2 in inflammation-induced ERS (Delmotte and Sieck, 2019). Downregulation of Mfn-2 aggravated ERS, attributing to the proliferation of airway smooth muscle.

A study from Sun et al. (2015) showed that miR-214 mediated ISO-induced proliferation and collagen synthesis in cardiac fibroblasts through directly targeting Mfn-2 and activating the downstream ERK1/2 MAPK signaling pathway.

## Mitofusin-2 and Cancer

Abnormal cell proliferation could lead to the destabilization of chromosomal and genetic organization, resulting in the formation of neoplasm. This effect is regulated by a series of genes. Mutation of the genes will drive cells into tumor cells. The increase in glucose uptake and enhanced glycolytic rates indicate that metabolic alteration provides growth advantages for tumor cells (Lunt and Vander Heiden, 2011). In addition, tumor cells may exert different metabolic ways from normal cell proliferation. Warburg noticed that tumor cells exhibit a high rate of glycolysis even in the presence of oxygen (aerobic glycolysis) (Ortega et al., 2009). Some types of cancer, such as bladder cancer, cervical cancer, and breast cancer, are associated with altered mitochondrial morphology and metabolism (Filadi et al., 2018). The expression level of Mfn-2 is usually decreased in various types of cancer, and increasing Mfn-2 expression can suppress cell proliferation (Rehman et al., 2012), indicating that Mfn-2 is a tumor suppressor. One mechanism by which Mfn-2 suppresses cancer cell proliferation is inhibiting the metabolic flux to aerobic glycolysis by interreacting with pyruvate kinase 2 (PKM2) *via* the N-terminus (Li et al., 2009); phosphorylation of Mfn-2 enhances this interaction. Rictor is a subunit of mTORC1; Rictor deletion could block mitochondrial OXPHOS. Xu’s recent work showed that Mfn-2 plays a vital role in the inhibition of the mTORC2/Akt signaling pathway through the interaction with Rictor by binding its HR1 domain in breast cancer patients. Mfn-2 knockdown could enhance growth in breast cancer. All these evidence indicated that the mTORC/AKT pathway is closely linked with Mfn-2 in pathological cell proliferation.

Under physiological conditions, mitochondria take up calcium mainly from the ER. Calcium is released through inositol 1,4,5-trisphosphate (IP3) receptors (IP3R) and ryanodine receptors (RyRs) on the ER membrane (Marks, 1997) and enters mitochondria through voltage-dependent anion channels (VDACs). The relationship between calcium signaling pathway and cancer has been discussed in detail in previous reviews (Ivanova et al., 2017; Marchi et al., 2018). In this process, MAM play a vital role (Ivanova et al., 2017) because these harbor key calcium handling proteins such as IP3R, VDACs, and sigma-1 receptors (Hayashi and Su, 2007). In 2008, de Brito and Scorrano (2008) showed that Mfn-2 was required for mitochondrial calcium uptake, and Mfn-2 knockout MEFs exhibited a reduced calcium uptake rate. In addition, translation of the calcium signals is necessary for the regulation of cell death and survival. Lower ER calcium content could arouse the expression of anti-apoptotic protein expression, such as Bcl-2 (Pinton et al., 2001). Mfn-2 could restore calcium homeostasis and downregulate ER stress in mouse neuroblastoma N2a cells.

Besides the regulation of intracellular calcium transportation, Mfn-2 may also regulate other signaling pathways for cell proliferation. Transcription factor SP1 plays an essential role in the expression of PITPNM3 in cancer cells, and Mfn-2 was reported to have anti-tumor activity by interacting with transcription factor SP1 directly (Tang et al., 2020). Pang et al. (2019) found that Mfn-2 inhibited cell proliferation *via* the Wnt/β-catenin pathway in bladder cancer. Mfn-2 knockdown increased the translocation of β-catenin into the nucleus, resulting in larger tumor volumes and a higher proliferation index. Pharmacological inhibition of the Mfn-2/mTORC2/Akt pathway attenuated tumor growth (Xu et al., 2017). Several other studies also indicated that Mfn-2 interacted with the proapoptotic Bcl-2 family members Bax and Bak (Suen et al., 2008; Li et al., 2020).

## Future Directions

Despite the significant advancements that deepened our understanding of the structure and functions of Mfn-2, several key questions remain to be elucidated:

(1) Although some evidence indicated that, in addition to fusion reaction, Mfn-2 has been reported to regulate cell proliferation *via* various signaling pathways, there are currently no studies that figured out whether these pathways are related to mitochondria fusion. More studies on this topic may be necessary in the future.
(2) Although there are many evidence about the role of Mfn-2 in neoplastic diseases, the microenvironment may change the impact of Mfn-2 in different types of cancers. In the future, a more detailed analysis of Mfn-2 may be necessary.
(3) Could altered Mfn-2 act as a potential biomarker in pathogenesis?

## Conclusion

The machineries of mitochondrial fusion/fission and the significance of mitochondrial dynamics in regulating mitochondrial and cellular functions have been established. The mitochondrial fusion protein Mfn-2 regulates mitochondrial morphology, metabolism, calcium homeostasis, and mtDNA stability. Mfn-2 also plays a role *via* MAM formation and mitophagy to regulate cell proliferation and cell survival/death in different tissues. Many important questions await extensive investigation. In the future, studies on the role of Mfn-2 in cell proliferation may lead to the development of new strategies for treating various diseases, such as cancer and cardiovascular diseases.

## Author Contributions

YX and JL draft the manuscript. WW revised and polished the manuscript. XL supervised the review and established the whole frame. All authors contributed to the article and approved the submitted version.

## Conflict of Interest

The authors declare that the research was conducted in the absence of any commercial or financial relationships that could be construed as a potential conflict of interest.
